# Current News

**Published:** 1899-10

**Authors:** 


### Current News.

### NORTHEASTERN DENTAL ASSOCIATION.

The Fifth Annual Meeting of the Northeastern Dental Association will be held in Hotel Hamilton, Holyoke, Mass., on Wednesday and Thursday, October 18 and 19, 1899. The Executive Committee hope for and desire a large attendance, as their labors have been crowned with such success that the entire exhibit-space has already been engaged. Essayists of prominence have accepted invitations enough to fill up all the time allotted; clinics enough have been promised, and thus a meeting worthy of your attendance has been arranged. Be sure and save out the above dates and attend. A cordial invitation is extended to the profession at large to attend.

EDGAR O. KINSMAN, D.D.S.,

*Secretary.*

### UNION MEETING, SEVENTH AND EIGHTH DISTRICT DENTAL SOCIETIES, STATE OF NEW YORK.

The Thirty-second Union Meeting of the above Societies will be held in the assembly-room of the New Osbourn House, Rochester, N. Y., Tuesday, Wednesday, and Thursday, October 24, 25, and 26, 1899.

WM. W. BELCHER,
*Chairman.*

827 GRANITE BUILDING, ROCHESTER, N. Y.

